# Comparative Genomics to Delineate Pathogenic Potential in Non-O157 Shiga Toxin-Producing *Escherichia coli* (STEC) from Patients with and without Haemolytic Uremic Syndrome (HUS) in Norway

**DOI:** 10.1371/journal.pone.0111788

**Published:** 2014-10-31

**Authors:** Kjersti Haugum, Jostein Johansen, Christina Gabrielsen, Lin T. Brandal, Kåre Bergh, David W. Ussery, Finn Drabløs, Jan Egil Afset

**Affiliations:** 1 Department of Laboratory Medicine, Children’s and Women’s Health, Faculty of Medicine, Norwegian University of Science and Technology, Trondheim, Norway; 2 Department of Cancer Research and Molecular Medicine, Faculty of Medicine, Norwegian University of Science and Technology, Trondheim, Norway; 3 Department of Foodborne Infections, Norwegian Institute of Public Health, Oslo, Norway; 4 Biosciences Division, Oak Ridge National Labs, Oak Ridge, Tennessee, United States of America; 5 Department of Medical Microbiology, St. Olavs University Hospital, Trondheim, Norway; Federal University of São Paulo, Brazil

## Abstract

Shiga toxin-producing *Escherichia coli* (STEC) cause infections in humans ranging from asymptomatic carriage to bloody diarrhoea and haemolytic uremic syndrome (HUS). Here we present whole genome comparison of Norwegian non-O157 STEC strains with the aim to distinguish between strains with the potential to cause HUS and less virulent strains. Whole genome sequencing and comparisons were performed across 95 non-O157 STEC strains. Twenty-three of these were classified as HUS-associated, including strains from patients with HUS (n = 19) and persons with an epidemiological link to a HUS-case (n = 4). Genomic comparison revealed considerable heterogeneity in gene content across the 95 STEC strains. A clear difference in gene profile was observed between strains with and without the Locus of Enterocyte Effacement (LEE) pathogenicity island. Phylogenetic analysis of the core genome showed high degree of diversity among the STEC strains, but all HUS-associated STEC strains were distributed in two distinct clusters within phylogroup B1. However, non-HUS strains were also found in these clusters. A number of accessory genes were found to be significantly overrepresented among HUS-associated STEC, but none of them were unique to this group of strains, suggesting that different sets of genes may contribute to the pathogenic potential in different phylogenetic STEC lineages. In this study we were not able to clearly distinguish between HUS-associated and non-HUS non-O157 STEC by extensive genome comparisons. Our results indicate that STECs from different phylogenetic backgrounds have independently acquired virulence genes that determine pathogenic potential, and that the content of such genes is overlapping between HUS-associated and non-HUS strains.

## Introduction

Shiga toxin producing *E. coli* (STEC) are important human pathogens known to cause infections ranging from diarrhoea and haemorrhagic colitis to haemorrhagic uremic syndrome (HUS) [1]. Since the first reports of disease caused by O157:H7 in 1982 [2], [3], this serotype has been the most frequently reported cause of severe STEC disease and outbreaks worldwide [1]. However, several non-O157 STEC serogroups (*e.g.* O26, O45, O103, O111, O121 and O145) have also been recognized to be responsible for severe disease and outbreaks [4], [5].

The STEC pathotype is defined by the presence of Shiga toxins Stx1 and Stx2 encoded by the *stx1* and *stx2* genes, which are acquired through horizontal gene transfer of a heterogeneous group of lambdoid bacteriophages [6]–[9]. There are several subtypes of Shiga toxins, of which the Stx2 subtypes Stx2a, Stx2c and Stx2d are more often associated with HUS than other Stx subtypes [10]–[13]. In addition, the adherence factor intimin, encoded by the *eae* gene located in the Locus of Enterocyte Effacement (LEE) pathogenicity island, is important for STEC pathogenicity. STEC causing severe disease and outbreaks do usually harbour LEE [1], [14], [15], although also LEE negative STEC are sometimes found in patients with severe disease [15]–[21]. LEE encodes several genes responsible for the attaching and effacing nature of STEC, a feature that these bacteria share with the closely related enteropathogenic *E. coli* (EPEC). In addition, the LEE encodes additional associated regulators, translocators, effector proteins and chaperones [22], [23].

Whole genome sequencing of bacterial genomes has become an accessible and affordable analysis. Comparison of whole genome sequences provides information on gene content and organization, and gives an overview of how organisms are related. Whole genome sequences available of STEC and other *E. coli* have demonstrated high diversity among different *E. coli* genomes, due to horizontal gene transfer, gene loss and other genomic alterations [20], [24]–[34]. Genomic comparisons of O157 and non-O157 LEE positive STEC genomes with other *E. coli* and *Shigella* have also revealed that LEE positive STEC in general have larger genomes, mostly due to horizontally transferred DNA such as prophage DNA, plasmids and integrative elements encoding potential virulence factors [24]–[26].

In Norway non-O157 STEC are more frequently isolated from patients with STEC disease than O157, and are also more common than O157 STEC in patients suffering from HUS [35]. Although whole genome sequence comparisons of O157 and non-O157 STEC are available [20], [25], [28], it is still unclear whether it is possible to differentiate between STEC strains based on their potential to cause HUS. In this study our main aim was to compare whole genome sequences from 95 non-O157 human STEC strains to investigate potential genetic differences suitable for distinguishing between highly pathogenic STEC having caused HUS and low-virulent STEC having caused only mild disease or asymptomatic carriage. We were not able to clearly distinguish between HUS-associated and non-HUS non-O157 STEC by extensive genome comparisons in this study. Our results indicate that STEC from different phylogenetic backgrounds have independently acquired virulence genes that determine pathogenic potential, and that the content of such genes is overlapping between HUS-associated and non-HUS strains. To our knowledge this is the largest collection of non-O157 STEC strains that has been sequenced to date, thus providing valuable data on the less characterized STEC serotypes.

## Results

Sequencing and whole genome comparison of the 95 non-O157 STEC strains included in this study revealed high degree of variation in gene content as well as diversity in whole genome phylogeny. A total of 1,954 genes represented the core genome among the 95 strains, while 26,073 genes represented the pan genome. The LEE pathogenicity island was identified in 54 (57%) of the genomes, whereas 41 (43%) of the sequenced STEC strains were LEE negative (Table 1). Stx genes were detected in 84 (88%) genomes; *stx1* in 35 (37%), *stx2* in 37 (39%) and a combination of *stx1* and *stx2* in 12 (13%) of the genomes (Table 1). Eleven (12%) of the sequenced genomes which did not harbour stx genes, were classified as STEC-LST (Table 1). The *stx2* subtypes were differently distributed: *stx2a* was significantly more frequent among LEE positive STEC, while *stx2b* was more frequent among LEE negative strains (p<0.05 for both analyses) (Table 1). Of the *stx1* subtypes, *stx1c* was significantly associated with LEE negative STEC.

**Table 1:** Distribution of *stx1*, *stx2* and their subtypes in 95 Norwegian non-O157 LEE positive and LEE negative STEC strains.

	LEE positive n = 54		LEE negative n = 41		Total n = 95		
Gene	n	(%)	n	(%)	n	(%)	p-value
***stx1***	21	38.9	14	34.1	35	36.8	ND
***stx2***	22	40.7	15	36.6	37	38.9	ND
***stx1+stx2***	4	7.4	8	19.5	12	12.6	ND
**STEC-LST** 1	7^2^	13.0	4^3^	9.8	11	11.6	ND
**stx1 subtype**							
***stx1a***	24	44.4	10	24.4	34	35.8	>0.05
***stx1c***	1	1.9	11	26.8	12	12.6	0.0003
***stx1d***	0	0.0	1	2.4	1	1.1	>0.05
**stx2 subtype**							
***stx2a***	24	44.4	5	12.2	29	30.5	0.00073
***stx2b***	0	0.0	14	34.1	14	14.7	0.0000017
***stx2c***	2	3.7	1	2.4	3	3.2	>0.05
***stx2d***	0	0.0	2	4.9	2	2.1	>0.05
***stx2e***	0	0.0	1	2.4	1	1.1	>0.05

1 STEC-LST: STEC that has lost Shiga toxin.

2 Six of these strains, which were stx2 negative *E. coli* when initially tested at the Norwegian Public Health Institute, had been isolated from a patient with HUS or had a MLVA profile identical to an outbreak STEC strain and was epidemiologically related to that HUS case. The last strain had been stx2 positive when initially tested, but had lost the stx-gene at a later stage.

3 When initially tested, three of these strains contained stx1 and one strain contained stx2.

In the present study, all 19 STEC strains from patients with HUS were from children <5 years old (Table S1 in File S1). An additional four strains were epidemiologically linked to a HUS-case, and consequently 23 strains were classified as HUS-associated (Table S1 in File S1). All the HUS-associated STEC strains harboured the LEE pathogenicity island, and except for six STEC-LST of serotype O103:H25 from the same outbreak, all contained the *stx2a* subtype. Only one STEC from a HUS patient (FHI6) harboured *stx1* (subtype *stx1a*), in addition to *stx2a* (Table S1 in File S1).

### Phylogenetic analysis of the core genome

**Figure 1:**
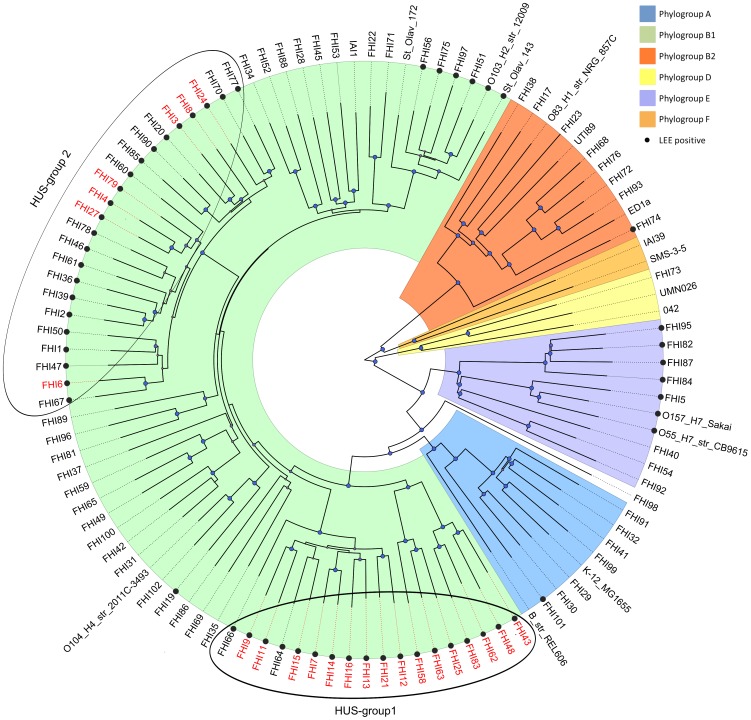
Core gene phylogeny of the 95 sequenced non-O157 STEC and 14 *E. coli* reference genomes. The tree was rooted in Figtree (http://tree.bio.ed.ac.uk/software/figtree/) by midpoint rooting. For an unrooted version of the phylogenetic tree, see Figure S1. The *E. coli* phylogroups are marked with the colours blue (A), green (B1), orange (B2), yellow (D), ochre (F) and indigo (E). Bootstrap values were scaled from 0–1, and blue circles indicate a bootstrap value of ≥0.8. LEE positive STEC were marked with •, while all HUS and HUS-associated STEC included in the study were indicated with red letters.

A core gene tree was constructed from alignment of the 1,861 core genes present in all the 95 STEC and 14 *E. coli* reference genomes representing the *E. coli* phylogroups (109 genomes in total). In this phylogenetic tree, the 95 strains were distributed in the *E. coli* phylogroups A, B1, B2, D and E (Figure 1). In general, clusters of LEE negative STEC strains were distributed between clusters of LEE positive strains. Most of the strains belonged to the B1 phylogroup, and a majority of the LEE positive STEC strains were also found within this group. All HUS-associated strains were found in phylogroup B1, in two clusters which we designated HUS-group 1 and 2 (Figure 1). The STEC strains in HUS-group 1 were distributed in three related clusters, consisting of mainly HUS and HUS-associated strains of serotypes O103:H25, O145:H[unknown], and O121:H- (n = 18) (Figure 1, Figure S1). HUS-group 2 consisted of one distinct cluster of strains (n = 23), in which all HUS and HUS-associated STEC strains of serogroups O26, O86 and O111 were located. Sixteen of the strains in HUS-group 2, of which 13 strains were of serogroup O26, were not associated with HUS (Figure 1, Figure S1). This group therefore appeared to be more heterogeneous than HUS-group 1 with respect to pathogenicity.

### Comparative analysis of the accessory genome

The accessory genome consisted of approximately 24,000 genes. PCA of the accessory genome separated LEE positive (n = 54, Table S1 in File S1) and LEE negative (n = 41) STEC strains in two distinct groups (data not shown). PCA and PLS regression of LEE positive strains (n = 54) as well as of LEE positive/*stx2* positive strains (n = 33, Table S1 in File S1) could not further separate the strains into subgroups. In a random forest analysis of the accessory genome in the LEE positive strains (n = 54) 18 of the 23 HUS-associated strains were correctly classified as HUS-strains, whereas 29 of the 31 non-HUS strains were correctly classified (Table S2 in File S2, Figure S2). However, a perfect separation of the two groups could not be achieved.

By comparison of all 54 LEE positive STEC strains, we identified eleven genes which were more frequent in the HUS-associated strains (n = 23) and four genes which were more frequent in non-HUS STEC strains (n = 31) (p<0.01, FDR) (Table 2, Table S3 in File S2). None of these genes were however present exclusively in one of these two groups. Among the 33 LEE positive STEC strains containing *stx2* (Table S1 in File S1), we identified 69 genes which were overrepresented in HUS-associated strains (n = 23) and 44 genes which were more frequent in non-HUS STEC strains (n = 10) (p<0.01, data uncorrected) (Table 2, Table S4 in File S2).

**Table 2:** Overview of the different subgroups of STEC that were compared in this study.

Genesource	Groups defined by	Groups of strainsthat were compared	Number of genesoverrepresented in group	False discoveryrate (FDR)	Number of genes or genevariants unique to group
**Accessory** **genome**	**Clinical and** **epidemiological** **information**	LEE+/*stx2+* HUS^1^ n = 23	11	≤0.01	0
		Other LEE+ non-HUS n = 31	4	≤0.01	0
		LEE+/*stx2+* HUS^1^ n = 23	69	≥0.01	0
		LEE+/*stx2+* non-HUS n = 10	44	≥0.01	0
	**Core gene** **phylogeny**	HUS-group 1 (LEE+) n = 18	357	≤0.01	1^2^
		LEE+ other than HUS-group 1 n = 36	365	≤0.01	0
		HUS-group 2 (LEE+) n = 23	576	≤0.01	4
		LEE+ other than HUS-group 2 n = 31	218	≤0.01	0
		LEE+ O26 HUS^1^ n = 5	17	≥0.01	0
		O26 non-HUS n = 13	13	≥0.01	0
**Core** **genome**	**Clinical and** **epidemiological** **information**	LEE+/*stx2+* HUS^1^ n = 23	281	≤0.01	0
		Other LEE+ non-HUS n = 31	0	≤0.01	0
	**Core gene** **phylogeny**	O26 *stx2+* n = 8	87	≥0.01	0
		O26 *stx1+* n = 10	83	≥0.01	1
		O26 HUS^1^ n = 5	84	≥0.01	0
		O26 non-HUS n = 13	78	≥0.01	0

In the upper half of the table, different groups were compared with respect to gene content in the accessory genome. In the lower half, the groups were compared with respect to gene variants in the core genome.

1 HUS: HUS-associated STEC.

2 The gene was not exclusive to this group as it was also found in one LEE negative STEC.

When STEC strains in HUS-group 1 (n = 18, Figure 1) were compared with all other LEE positive strains (n = 36), 357 genes were more frequent in HUS-group 1 strains (p<0.01, FDR) (Table 2, Table S5 in File S2). One gene encoding a hypothetical protein (Table S5 in File S2) was present in all strains in HUS-group 1 but absent in other LEE positive STEC. This gene was however present in one LEE negative strain. When STEC strains in HUS-group 2 (n = 23) were compared with all other LEE positive STEC strains (n = 31), 576 genes were overrepresented in the former group (p<0.01, FDR) (Table 2, Table S6 in File S2). Four genes were present in all strains in HUS-group 2 while absent in the other strains (Table 2, Table S6 in File S2). Seventeen genes were overrepresented in HUS-associated serogroup O26 strains in HUS-group 2 (n = 5), whereas 13 genes were more frequent in the non-HUS strains of the same serogroup (n = 13) (p<0.01, uncorrected) (Table 2, Table S7 in File S2).

### Comparative analysis of the core genome

Analysis of the core genome identified in total approximately 13,000 gene variants by edit distance analysis. Different Pfam domains, and therefore different protein sequences, were observed in 13 of these gene variants (Table S8 in File S2). Comparison of core gene variants among LEE positive STEC (n = 54, Table S1 in File S1) identified 281 gene variants that were overrepresented in the HUS-associated (n = 23) compared to non-HUS strains (n = 31) (p<0.01, FDR) (Table 2, Table S9 in File S2). None of the gene variants were however found only among HUS-associated strains.

PLS regression of the core gene variants in serogroup O26 strains in HUS-group 2 discriminated *stx2* positive O26 (n = 8) from *stx1* positive O26 strains (n = 10) (data not shown). Eighty-seven gene variants were more frequent in *stx2* positive O26 strains compared to *stx1* positive strains of the same serogroup (p<0.01, data uncorrected) (Table 2, Table S10 in.

File S2), but none of these gene variants were exclusive for the group of *stx2* positive strains. Eighty-four gene variants were more common in the O26 strains which were HUS or HUS-associated (n = 5) compared to the O26 non-HUS strains (n = 13) (p<0.1, data uncorrected) (Table 2, Table S11 in File S2), but also in this case none of the variants were exclusive to either group.

### Comparison of HUS and non-HUS STEC from specific outbreaks

Comparative analysis on gene content was furthermore performed on specific HUS and non-HUS STEC that were epidemiologically linked and belonged to the same MLVA (Multiple Loci VNTR Analysis) outbreak cluster (Table S1 in File S1). In HUS-strain FHI4 (Table S12 in File S3) we identified 179 genes (Table S13 in File S3) which were absent in the non-HUS strain FHI3 (Table S12 in File S3) from the same outbreak. The majority of the genes were related to various mobile genetic elements integrated in the bacterial chromosome, Nle effectors (Table S13 in File S3) or plasmid pO26_1 (AP010954) (Table S13 in File S3). A closer search revealed additional plasmid genes on other contigs in the FHI4 draft genome, indicating the presence of a complete pO26_1 plasmid in this strain, while the corresponding genes were not found in strain FHI3. In HUS-strain FHI48 we identified 153 genes (Table S14 in File S3) that were absent in the non-HUS strains FHI43 and FHI62 from the same outbreak cluster. Again, most of the genes were related to mobile genetic elements (Table S14 in File S3). In the two HUS strains FHI58 and FHI63 from another outbreak, we identified 54 genes exclusive to these two strains (Table S15 in File S3), while another 506 genes were present only in the non-HUS strain St. Olav104. The genes in the two HUS strains were related to various functions, while in the non-HUS strain, the majority of the genes were related to mobile genetic elements and several Nle effectors (Table S15 in File S3).

### Gene ontology enrichment analysis

Gene ontology (GO) analysis of genes significantly overrepresented in the 23 HUS-associated STEC strains (Table S3 in File S2) revealed that nine GO terms in biological processes were enriched in these strains. The enriched terms specified metabolic and catabolic processes related to degradation of L-idonate (GO:0046183) (Table S16 in File S4).

Among the 357 genes more frequent in HUS-group 1 (Table S5 in File S2), we identified six enriched GO terms, in biological processes (n = 4), molecular functions (n = 1) and cellular components (n = 1) (Table S17 in File S4). Also in this case we identified enrichment in GO terms related to degradation of L-idonate (GO:0046183, GO:0003939, GO0019523). In biological processes and cellular components, we furthermore identified enriched GO terms related to protein secretion by the type II secretion system (GO:0015628, GO:0015627) (Table S17 in File S4).

Twenty-six GO terms were enriched in HUS-group 2 (Table S6 in File S2); in biological processes (n = 11), molecular function (n = 4) and cellular components (n = 11) (Table S18 in File S4). In biological processes, enriched terms were for siderophore biosynthetic process (GO:0019290) and ciliary or bacterial-type flagellar motility (GO:0001539) (Table S18 in File S4). For molecular function we identified enrichment in terms for motor activity (GO:0003774), isochorismate synthase activity (GO:0008909) and oxo-acid-lyase activity (GO:0016833), while for cellular components we found enrichment in terms for bacterial-type flagellum basal body and rod (GO:0030694) (Table S18 in File S4). None of the enriched GO terms were however unique for the HUS-associated STEC strains.

### Accession numbers

The nucleotide sequences are submitted to the European Nucleotide Archive with accession numbers ERS480133–ERS480228. Study accession number is PRJEB6447.

## Discussion

In this study we have performed comparative genomic analyses on what to our knowledge is the largest collection so far of genome-sequenced non-O157 STEC strains, in order to investigate if there were genetic differences suitable for distinguishing between highly pathogenic STEC having caused HUS and low-virulent STEC having caused only mild disease or asymptomatic carriage. Whole genome sequencing and comparison revealed that there was considerable heterogeneity in genetic content across the 95 non-O157 STEC strains included in the study. The approximately 24,000 genes constituting the accessory genome contribute to this heterogeneity, while 1,954 core genes were shared by all the sequenced strains. Much of the accessory genome contained various mobile genetic elements, which have also previously been shown to contribute to heterogeneity and pathogenic evolution in *E. coli* [24]–[26], [30], [36]. The results from principal component analysis of the accessory genome where LEE positive strains were separated from LEE negative strains, is in line with several previous reports [14], [20], [28], [37]–[42]. Although the accessory genome was not identical within LEE positive STEC strains, further PCA analysis of LEE positive strains showed scattering of the strains without any distinct subgroups. Furthermore, although random forest analysis showed a slightly better classification than PCA, it could not classify all strains correctly, indicating that variable accessory gene content was heterogeneously distributed within this group.

The various *stx1* and *stx2* subtypes were differentially distributed between LEE positive and LEE negative STEC strains, i.e. *stx2b* and *stx1c* were more frequent among LEE negative strains (p<0.05) while *stx2a* was more frequent among LEE positive strains (p<0.05) (Table 1). All HUS associated STEC in this study were LEE positive and contained *stx2a*, except for the STEC-LST (strains that have lost Shiga toxin). Thus our results are in accordance with previous studies where Stx2a has been shown to possess higher potency than Stx1 and other Stx2 subtypes [43], and that LEE positive and *stx2a* positive STEC strains are more often associated with severe disease [12]–[15], [43]. Furthermore, all the HUS-associated STEC belonged to *E. coli* O serogroups known to be associated with STEC disease (Table S1 in File S1) [1]. However, although *stx2a* and LEE were typical for the HUS-associated STEC, these characteristics are not unique for such STEC, and thus not sufficient to clearly distinguish HUS-associated from non-HUS STEC. We therefore aimed to compare the accessory genomes of *stx2a/*LEE positive STEC in an attempt to differentiate between HUS-associated and non-HUS strains. This analysis revealed that certain genes were overrepresented among HUS-associated STEC (Table 2, Tables S3, S4 in File S2), suggesting that strains with this gene profile have a high pathogenic potential. However, none of the genes were exclusive for these strains, which further suggest that the gene content in HUS-associated STEC at least in part is shared with non-HUS STEC strains, or that different HUS strains have different gene content.

Core genome phylogeny revealed that the 95 non-O157 STEC strains were distributed over all the *E. coli* phylogroups except phylogroup F, confirming that the strains included in this study were heterogeneous. The majority of strains belonged to phylogroup B1 (Figure 1). Most of the LEE positive and all the HUS-associated strains in this study also clustered in this phylogroup, similarly to what has been reported in previous studies [15], [28], [31], [33], [44]. In addition, LEE negative STEC associated with HUS often belong to this phylogroup [20], [33], including the O104:H4 strain (FHI102) related to the 2011 German outbreak, which did however not cluster with any of the HUS and HUS-associated STEC strains in this study (Figure 1). LEE negative and LEE positive STEC did not form separate phylogenetic clusters, but were mixed in small clusters within several phylogroups as previously reported [20]. This indicates that the LEE pathogenicity island has been independently taken up by different STEC lineages at different time points. Because HUS-associated O103, O121 and O145 strains were distributed in three related clusters in the phylogenetic analysis, these STEC strains were classified as HUS-group 1, although they did not belong to one defined cluster. The remaining HUS-associated strains were located in one cluster which we termed HUS-group 2. This clustering of HUS-associated strains based on variation in core genes as observed in this study indicates that the phylogenetic backgrounds of the bacteria at least to some extent determine the pathogenic potential of the organism. In an attempt to search for unique genes in these groups, we analysed the accessory genome and identified several hundred genes that were significantly overrepresented in both groups, suggesting that different sets of genes may contribute to the pathogenic potential in different phylogenetic STEC lineages. However, few of these genes were found to be unique to any of the groups (Table 2, Tables S5, S6 in File S2), which further suggest that the accessory genome is shared both between and within the different clusters defined by the core genome phylogeny.

The majority of strains in HUS-group 2 were of serotype O26, of which HUS-associated strains clustered with non-HUS strains, suggesting that accessory factors rather than core genes defines pathogenic potential within this group. Regardless, it was not possible to identify any genes in the accessory genome which could reliably distinguish HUS-associated from non-HUS strains of the same serogroup in HUS-group 2 (Table 2).

In the core genome of the 95 STEC strain included in this study, we identified approximately 13,000 different gene variants by edit distance analysis. However, despite the high number of gene variants, differences in protein sequences were identified for only 13 of these variants. Comparison of the core gene variants revealed that although 281 gene variants were overrepresented in HUS-associated STEC, several of these were also present in strains not associated with HUS (Table 2, Table S9 in File S2). The observation that none of the identified gene variants were unique to HUS-associated STEC is supported by the fact that HUS-associated strains clustered to more than one group in the core gene phylogeny. Also for the O26 strains, although PLS regression revealed that serogroup O26 strains in HUS group 2 containing *stx2* were separated from strains that contained *stx1*, no core gene variants were found to be significantly over- or underrepresented in either of these two groups (Table 2, Table S10 in File S2).

By comparing the genomes of the STEC strains which were epidemiologically linked and belonged to the same MLVA outbreak cluster (Table S1 in File S1), we identified a number of genes that were different across HUS and non-HUS strains (Table S12 in File S3). The fact that different genes were present in strains from the same outbreak might indicate that the infecting source consisted of a mixture of similar but not identical STEC which could have evolved from the same clone. Regardless, we could not identify any genes that clearly distinguished between the HUS and non-HUS associated strains within each of these outbreak clusters.

GO terms related to L-idonate degradation were found to be enriched both among all 23 HUS-associated STEC strains collectively and the strains in HUS-group 1. *E. coli* is able to utilize L-idonate as a sole carbohydrate source through the Entner-Doudoroff metabolic pathway, which has been shown to be important for the ability of *E. coli* to colonize mammalian intestines [45]. In addition, we identified enriched GO terms for protein secretion by the type II secretion system in HUS-group 1. The type II secretion system in Gram negative bacteria promotes protein transport across the outer membrane, and the majority of proteins exported by this system contribute to bacterial adaptation and colonization by generating nutrients available for uptake [46]. Furthermore, certain exported lipoproteins have been shown to be involved in biofilm formation in EPEC [47]. Genes responsible for the enriched GO terms could therefore contribute to enhanced bacterial colonization and adaptation, which might have an impact on bacterial virulence in these specific strains. This however needs to be confirmed in further investigations. Of the 26 GO terms that were enriched in HUS group 2, a few were related to flagellar motility, which in general are recognized as virulence factors in bacteria [48]. In addition, enriched GO terms were related to siderophore biosynthesis. Siderophores, being iron chelating compounds, are important for iron acquisition in bacteria [49], [50]. The specific siderophore identified among strains in HUS-group 2 was encoded on a high-pathogenicity island (HPI) found in a distinct clonal lineages of STEC, which includes serogroup O26 [51], [52]. These results indicate that both motility and iron acquisition might be important factors for bacterial virulence of STEC in HUS-group 2. However, the precise role of these genes for STEC pathogenesis needs to be explored in further studies.

In addition to bacterial factors, it is clear that infection dose and host factors like the immune system, expression of the Shiga toxin receptor and intestinal environment might also affect STEC virulence, and thus severity of STEC disease [53]. In this study, all patients with HUS were <5 years old, which is known to be a risk factor for severe STEC disease [54], [55]. Unfortunately we could not obtain further information on host factors, but it is possible that such factors play an important part in explaining why highly similar strains lead to such different clinical outcomes in different patients.

In our study we included all non-O157 STEC strains from HUS-patients in Norway. However, these represent only a limited number of STEC strains from each phylogenetic lineage or serotype. Furthermore, few epidemiologically linked HUS and non-HUS STEC strains were included in the study. For future studies, if more STEC strains associated with HUS were included in the genomic comparisons this would give more strength both to phylogenetic and to statistical analyses. In addition, even if highly virulent STEC strains share overlapping genetic content with less pathogenic strains, further investigations regarding factors regulating transcription and translation as well as transcriptomics and proteomics analyses could shed further light into STEC virulence and pathogenicity.

## Conclusion

In this study whole genome sequencing and comparisons of 95 non-O157 STEC strains revealed that there were considerable genetic and phylogenetic heterogeneity between the strains. Although all HUS-associated STEC strains belonged to the B1 phylogroup, all non-O157 STEC from HUS patients did not cluster together, but were found in two separate clusters within this phylogenetic group. A clear difference in gene profile was observed between LEE positive and LEE negative STEC. A number of accessory genes were found to be significantly overrepresented among HUS-associated STEC, but none of them were unique to this group of strains. Our results indicate that STEC from different phylogenetic backgrounds independently have acquired virulence genes that determine pathogenic potential, and that specific genes overrepresented among HUS strains are not necessarily shared by all such strains, but that different sets of genes may contribute to the pathogenic potential in different phylogenetic STEC lineages.

## Materials and Methods

### Bacterial strains and clinical information

We selected 94 non-O157 STEC strains from the strain collection at the Norwegian Institute of Public Health (Oslo, Norway) isolated in 2000–2012 for sequencing in this study (Table S1 in File S1). In addition, three STEC strains (St. Olav104, St. Olav143 and St. Olav172, Table S1 in File S1) were selected from the strain collection at St. Olavs Hospital (Trondheim, Norway). The strains included in the study were primarily selected to represent different MLVA genotypes [56], [57], a diversity of non-O157 STEC serotypes and patients with different severity of disease (Table S1 in File S1). All available non-O157 STEC strains isolated from patients with HUS (n = 20) in Norway were included, except one strain (FHI10) which after whole genome sequencing was shown to be contaminated (Table S1 in File S1). Thus, a total of 96 strains were included in the study (Table S1 in File S1).

Some of the STEC strains from patients with HUS were from outbreaks and therefore had identical MLVA-genotypes or belonged to the same MLVA-genotype clusters (Table S1 in File S1). Four of the STEC strains included were furthermore classified as HUS-associated because they had identical MLVA-genotype as or belonged to the same MLVA-genotype cluster as a HUS case (Table S1 in File S1). Five of the STEC strains were from non-human sources and were isolated during various outbreak investigations related to STEC disease (Table S1 in File S1). One of these was designated as HUS-associated (FHI16, Table S1 in File S1). Of the total 96 STEC strains included in the study, 95 strains were included for genomic comparison throughout the whole study whereas one strain (St. Olav104) was included for parts of the study only. In addition, 14 *E. coli* were included as reference strains for classification of the STEC strains into the *E. coli* phylogroups A, B1, B2, D, E, and F (Table S1 in File S1).

Primary characterization of STEC at the Norwegian Institute of Public Health and St. Olavs Hospital had been based on PCR for the *stx1*, *stx2* and *eae* genes [58]–[60]. Ninety-one strains then had contained the *stx1* and/or *stx2* genes, while six strains of serotype O103:H25 did not have *stx* genes at inclusion time (Table S1 in File S1). The six *stx* gene negative O103:H25 strains were included in the study because they were isolated from patients with HUS in an outbreak (five strains), or was isolated from fermented sausage linked to this specific outbreak (one strain) [61] (Table S1 in File S1). In this outbreak, *stx2a* was detected in only two of the isolated strains. As *stx* negative derivates of STEC causing HUS occasionally are shed by HUS patients, the six strains without *stx* genes were regarded as STEC that had lost their *stx* genes, often termed EHEC/STEC-LST [62]. Fifty-five of the STEC strains were positive for the LEE pathogenicity island, as detected by the presence of the *eae* gene.

Serotyping was performed at the National Reference Laboratory for Enteropathogenic Bacteria at the Norwegian Institute of Public Health, using monospesific O:K and H antisera by a combination of in-house antisera before 2002, and by antisera from Sifin (Germany) and SSI (Denmark) after 2002, covering altogether 44 O-serogroups including O26, O103, O111, O121, O145, O157; and 8 H-antigens. Twenty-four of the strains included in the study did not belong to any of the serogroups tested for (Table S1 in File S1).

### Ethics Statement

This experimental study was approved by the Regional Committee for Medical and Health Research Ethics, REC South-East (REC number 2011/2314). Clinical data (including age and gender) required for classification of patients into the groups HUS, bloody diarrhoea, diarrhoea and no disease were obtained from Norwegian Surveillance System for Communicable Diseases (MSIS) at the Norwegian Institute of Public Health (Table S1 in File S1). Dispensation from professional secrecy requirements was given by the REC. As data were analysed anonymously informed consent was not obtained.

### DNA isolation

Strains were grown overnight on MacConkey agar. Genomic DNA was isolated for each strain using the Qiagen MagAttract DNA Mini M48 Kit and the Qiagen BioRobot M48 (Qiagen, Hilden Germany) as described by the manufacturer.

### Whole genome sequencing

Ninety-six of the STEC strains were sequenced with the Illumina Technology, while one strain (St. Olav104) was sequenced with Pacific Biosciences (PacBio) Technology (Table S1 in File S1).

For the strains to be sequenced by Illumina technology a standard read library of bacterial genomic DNA was prepared, with an average fragment length of 370 base pairs (bp). The DNA was sequenced by LGC Genomics (Berlin, Germany) on the Illumina HiSeq2000 platform (Illumina, San Diego, CA, USA) with 100 bp paired-end reads. Assembly and scaffolding of processed and error corrected paired-end reads was done using Velvet 1.2.04 [63]. Information on the resulting draft genome for each strain is given in Table S1 in File S1.

Forty-eight of the 96 strains were selected for additional mate pair sequencing (Table S1 in File S1). For this purpose a 2 kb Illumina Mate Pair library was prepared and the DNA was sequenced by LGC Genomics (Berlin, Germany) on the Illumina HiSeq2000 platform with 100 bp paired-end reads. Assembly and scaffolding of processed and error corrected paired-end reads was performed using Allpaths-LG release 45553 [64]. Gap closure of assembly scaffolds was done using SOAP GapCloser version 1.12 [65], while refinement of gap-closed scaffolds was done using SEQuel version 1.0.2 [66]. Information on the resulting draft genome for each strain is given in Table S1 in File S1.

Genome sequencing on the PacBio platform was performed at the Norwegian Sequencing Centre (Oslo, Norway). A library was prepared using the Pacific Biosciences 10 kb library preparation protocol, and size selection of the final library was performed using Ampure beads. The library was sequenced on a Pacific Biosciences RS II instrument (Pacific Bioscience, Menlo Park, CA, USA) using P4-C2 chemistry and three SMRT cells. Processed reads were assembled using HGAP v2 [67]. Information on the resulting draft genome is given in Table S1 in File S1.

### Gene annotation

Identification of open reading frames was performed using the Prodigal Microbial Gene Prediction Software [68], and functional gene annotation was done using myRAST [69].

### Comparative analyses

The CMG-biotools (Comparative Microbial Genomics) package was used for genome comparison [70]. Blastmatrix in CMG-biotools was used to identify proteins shared between genomes, while pancoreplot was used to identify the pan- and core-genome of the sequenced strains. In this context, genes were considered to be homologs having at least 90% sequence identity over at least 60% alignment length. The accessory genome was defined by subtracting all core genes from the pan genome. Genome analysis and comparison was performed across all sequenced STECs (Table S1 in File S1) except strain St. Olav104 which was only used for comparison with two HUS-strains (FHI58 and FHI63).

### Core genome phylogeny

*E. coli* phylotypes were determined *in silico* based on a core gene tree. This was created as described by Kaas et al. [33] using 1,861 core genes present in all the 95 STEC genomes and additional 14 *E. coli* reference genomes (109 genomes in total) representing the *E. coli* phylotypes A, B1, B2, D, E and F (Table S1 in File S1) [28], [33], [71]–[73].

### Core gene analysis

Core gene nucleotide sequences (n = 1,861) from the 95 STEC and 14 reference *E. coli* (Table S1 in File S1) were aligned separately and a consensus sequence was estimated for each of the 1,861 genes using EMBOSS 6.3.1 [74]. A python implementation of the edit distance method [75] was used to quantify the difference between the consensus sequence and the corresponding sequence of each core gene for all 109 strains included in the analysis. This resulted in various edit distances, representing different gene variants for each of the core genes. Edit distance values for all strains were normalized and transformed into a binary matrix for core gene comparisons.

To examine if any gene variant from the same core gene family showed different Pfam domains, we used pfam_scan.pl with the HMMER3 library of Pfam domains.

### Principal component analysis and Partial least squares regression

For Principal component analysis (PCA) and Partial least squares (PLS) regression the Laydi software (http://www.laydi.org) (unpublished) was used. For PLS regression, dependent variables (for the Y-matrix) were the clinical diagnosis HUS or classification as HUS-associated, and the presence of *stx1* and/or *stx2*. HUS and HUS-associated STEC-LST were classified as *stx2* positive for these analyses.

### Random forest analysis

Random forest analysis was performed using the randomForest package in R software package version 3.03 (R: A Language and Environment for Statistical Computing, http://www.R-project.org).

### Functional annotation and Gene Ontology enrichment analysis

Blast2GO was used for functional annotation based on gene ontology (GO) and for GO enrichment analysis [76], [77].

### Subtyping of Stx1 and Stx2

There are three known subtypes of Stx1 and seven known subtypes of Stx2, designated Stx1a, Stx1c and Stx1d, and Stx2a through Stx2g, respectively. Reference protein sequences were downloaded for each Stx subtype and Stx type variant from GenBank [13]. Amino acid sequences of the A and B subunits were concatenated and aligned separately for Stx1 and Stx2 using Clustal O in Jalview [78], [79]. For cluster analysis and tree calculations the Neighbour Joining algorithm in Jalview using % identity was used. Clustering of the Stx protein sequences of the sequenced strains with reference sequences was used to classify the former into Stx1 and Stx2 subtypes.

### Statistical analysis

Fisher’s exact test was used to analyse if specific *stx* subtypes were differently distributed in LEE positive and LEE negative STEC, with a p-value ≤0.05 regarded as statistically significant. Fisher’s exact test was also used to test if specific genes in the accessory genome were overrepresented, and for overrepresentation of gene variants in the core genome, in subgroups of the 95 STEC strains. Classification of the strains into subgroups was based on clinical and outbreak investigation information, phylogenetic analysis, and PCA and PLS regression (Table 2). For corrections of false discovery rate (FDR) in multiple testing the Benjamini-Hochberg method was used, with FDR≤0.01 regarded as statically significant. Whenever no significant association was identified after FDR correction, results for uncorrected analysis are given. The statistical analyses were performed using the R software package version 3.03 (R: A Language and Environment for Statistical Computing, http://www.R-project.org). In addition, in Blast2GO, Fisher’s exact test was used for GO enrichment analysis.

## Supporting Information

Figure S1 Unrooted core gene phylogeny. Unrooted core gene tree of the 95 sequenced non-O157 STEC and 14 *E. coli* reference genomes. The *E. coli* phylogroups are marked with the colours blue (A), green (B1), orange (B2), yellow (D), ochre (F) and indigo (E). LEE positive STEC were marked with a #-sign, while all HUS and HUS-associated STEC included in the study were coloured with red letters. HUS-group 1 consisted of 18 STEC strains in three related clusters, mainly strains of serotypes O103:H25, O145:H[unknown], and O121:H-. HUS-group 2 consisted of 23 STEC strains in one cluster, mainly strains of serogroups O26, O86 and O111.
(TIFF)

Figure S2 Heat map of the random forest classification of accessory genes in HUS- and non-HUS-strains. The one hundred genes that contributed most to the random forest classification were included. In the upper solid coloured line, HUS-strains are defined by blue colour and non-HUS-strains with red colour. Genes present are indicated by beige colour, while genes absent are indicated by red colour. In this classification, two HUS-strains were incorrectly classified as non-HUS-strains, while three non-HUS-strains were incorrectly classified as HUS-strains.
(TIFF)

File S1 Contains Table S1. Information on 95 Norwegian non-O157 STEC genomes sequenced and analysed in this study.
(XLSX)

File S2 Contains Tables S2–S11. Statistical analysis of accessory and core genes in the 95 sequenced Norwegian non-O157 STEC strains.
(XLSX)

File S3 Contains Tables S12–S15. Analysis of genes present in HUS- and non-HUS-strains from the same outbreaks.
(XLSX)

File S4 Contains Tables S16–S18. Gene ontology analysis of genes overrepresented in HUS-associated strains.
(XLSX)
