# Supplementary figures and images for: Comparative Genomics to Delineate Pathogenic Potential in Non-O157 Shiga Toxin-Producing Escherichia coli (STEC) from Patients with and without Haemolytic Uremic Syndrome (HUS) in Norway
Source: PLoS One. 2014 Oct 31;9(10):e111788. doi: 10.1371/journal.pone.0111788 (PMC4216125; doi:10.1371/journal.pone.0111788)

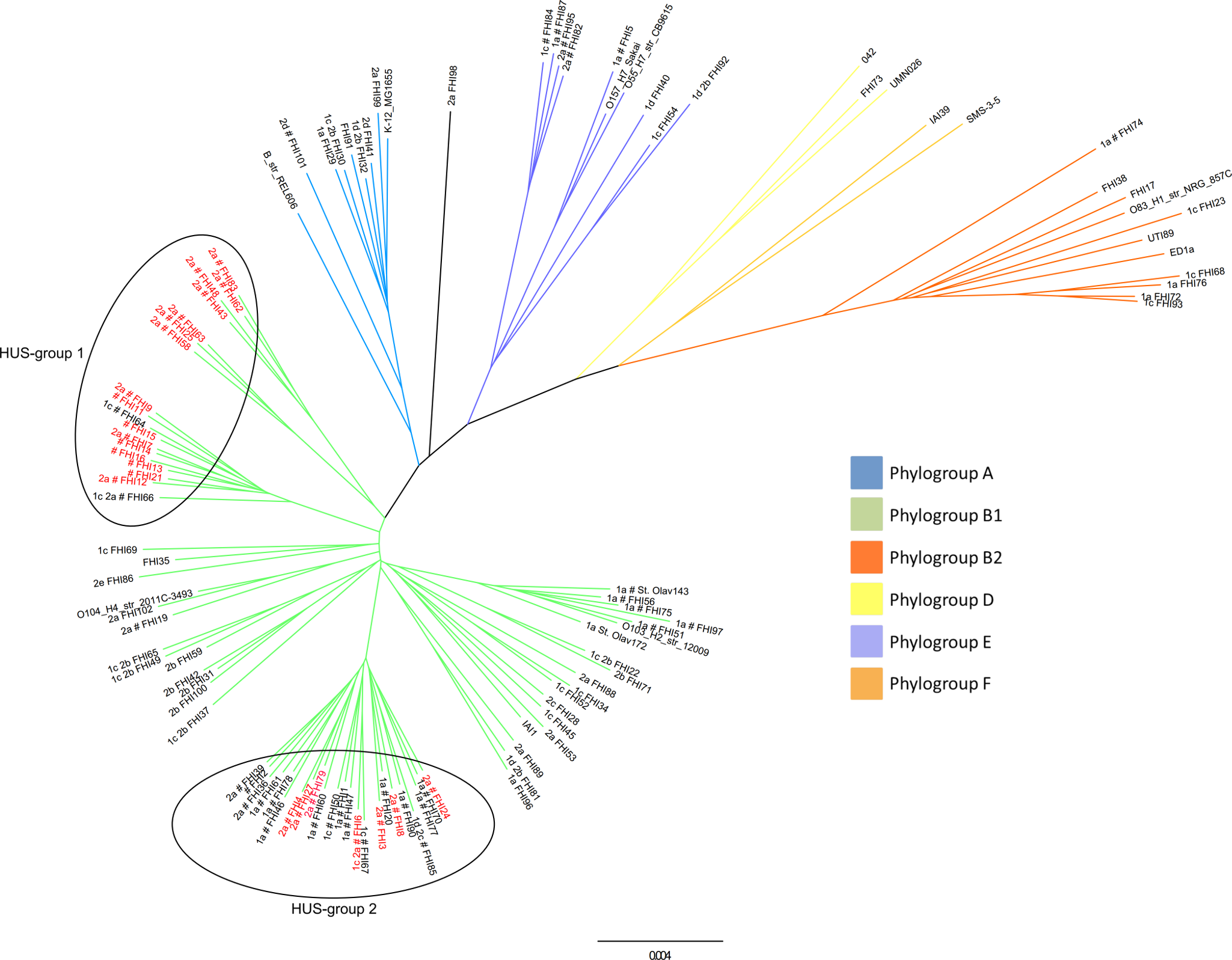

Supplement: Figure S1 — Unrooted core gene phylogeny. Unrooted core gene tree of the 95 sequenced non-O157 STEC and 14 E. coli reference genomes. The E. coli phylogroups are marked with the colours blue (A), green (B1), orange (B2), yellow (D), ochre (F) and indigo (E). LEE positive STEC were marked with a #-sign, while all HUS and HUS-associated STEC included in the study were coloured with red letters. HUS-group 1 consisted of 18 STEC strains in three related clusters, mainly strains of serotypes O103:H25, O145:H[unknown], and O121:H-. HUS-group 2 consisted of 23 STEC strains in one cluster, mainly strains of serogroups O26, O86 and O111. (TIFF) [file pone.0111788.s001.tiff]
